# Drying Kinetic of Jaboticaba Berries and Natural Fermentation for Anthocyanin-Rich Fruit Vinegar

**DOI:** 10.3390/foods12010065

**Published:** 2022-12-23

**Authors:** Lee Suan Chua, Nurul Syafiqah Abd Wahab

**Affiliations:** 1 Department of Bioprocess and Polymer Engineering, Faculty of Chemical and Energy Engineering, Universiti Teknologi Malaysia, UTM Skudai, Johor Bahru 81310, Malaysia; 2 Institute of Bioproduct Development, Universiti Teknologi Malaysia, UTM Skudai, Johor Bahru 81310, Malaysia

**Keywords:** jaboticaba, drying, thin-layer model, fruit vinegar, fermentation, anthocyanins

## Abstract

This study aimed to determine the drying kinetic of jaboticaba berries that were then used for the fermentation of natural fruit vinegar. The drying behavior was fitted well to the thin-layer kinetic model of Midilli et al. in a vacuum oven at 40 °C. Moisture diffusion was the dominant mechanism because two falling rate periods were observed. The effective moisture diffusivity was decreased (2.52 × 10^−10^ m^2^/s) after being pretreated with 70% sugar (1.84 × 10^−10^ m^2^/s) and 10% salt (6.73 × 10^−11^ m^2^/s) solutions. Fresh berry vinegar was found to have higher flavonoids, including anthocyanins, to exhibit higher antiradical and anti-pathogenic microorganism activities. However, the phenolic content in dried berries vinegar was higher, mainly from the bond breaking of tannins and lignins from fruit peel. Some extent of oxidation occurred because of the change in the color index of vinegar samples. The acidity of both vinegars was 3% acetic acid. Headspace GC-MS also detected acetic acid as the major compound (>60%) in the vapor of vinegar samples. A wide range of non-volatile compounds composed of alkaloids, terpenoids, flavonoids, organic acids, and sugar derivatives was detected by UHPLC-TWIMS-QTOFMS. The peak intensity of anthocyanins was reduced by 28–77% in dried berry vinegar. Therefore, it is better to prepare natural fruit vinegar using fresh berries, preserving anthocyanins for high antioxidant capacity.

## 1. Introduction

Fruits and vegetables are traditionally dried under the sun for preservation. However, this technique is weather-dependable, time-consuming, and also prone to contamination. The main purpose of food drying is to reduce the water content to prevent microbial growth, which will consequently diminish product quality during storage. The common drying techniques include vacuum drying, freeze drying, osmotic drying, and a combination of different drying technologies to achieve the desired moisture level. Often, thermal drying is the commonly used drying technique to accelerate the drying rate, mainly for energy saving. On the other hand, high-temperature drying always changes the sensory and nutritional level of fruits and vegetables, in addition to the irreversible change of their appearance and structure. Stamenkovic et al. [1] reported the loss of 32–40% total phenols, 3–25% flavonoids, 44–60% anthocyanins, and 0.94–97.9% vitamin C in dried raspberries during conventional convective drying.

Osmotic dehydration is incorporated into the conventional convective drying system as a pretreatment. It is applied to partially remove water from sample matrices by immersing them in a highly concentrated osmotic solution, mostly sugar and salt [2]. The introduction of the pretreatment improves energy saving in convective drying. Moreover, the pretreatment increases the ratio of sugar and acid in fruits, thus enhancing the organoleptic sensation of dried fruits, particularly for highly acidic fruits [3]. The diffusion of moisture from fruit matrices occurs as a result of osmotic pressure, and the dissolved solutes are simultaneously diffused from the highly concentrated osmotic solution into sample matrices. In addition to water, other small molecules such as minerals and vitamins from fruits may also diffuse into the osmotic solution. Therefore, the quality of dried fruits shall be monitored to minimize the physical and chemical changes while preserving fruits for shelf life extension. This can be achieved by applying an appropriate drying kinetic model, which is strongly affected by the nature of samples and drying technology.

Thin-layer drying model is widely used by researchers to determine the drying kinetics of fruits and vegetables. Samples are fully exposed to a temperature controlled airstream, and the temperature is assumed to be uniformly distributed to the thin structure of fruits or vegetables that have been sliced before drying. The thin-layer model can be also applied to the materials with multilayer of thicknesses, provided that the drying temperature and the relative humidity of the drying air are in the same thermodynamic condition at any time of the drying process. It is important to estimate the drying kinetics of products in order to understand their drying behavior from the experimental data and eventually optimize the energy requirement in drying. There is no single thin-layer model to effectively generalize the drying kinetics of all kinds of fruits and vegetables. This is because many factors including drying methods, drying conditions, and the product to be dried affect the drying rate. Previously, the Lewis model was likely to be the best model fitting to the kinetic data of drying jaboticaba pulp using a forced air oven at 50 °C and 60 °C [4]. A 10 °C increment in drying temperature shortened the drying time from about 18 h to 9 h. Tai et al. [5] reported that Page model was the best fit model to the experimental data of drying banana peel. However, Bolaji et al. [6] found that the two-term model well explained the drying behavior of maize. The works of Mugodo et al. [7] proved that the model of Midilli et al. was suitable to explain the drying behavior of mango slices in convective oven drying, uncontrolled solar drying, and modified ventilation greenhouse solar drying techniques. With almost similar drying techniques, logarithmic model was found to fit to the drying data of okra slices [8]. Therefore, it is important to investigate the drying kinetic of individual sample matrices in different drying systems in order to understand the underlying drying mechanism.

*Plinia cauliflora* is a Brazilian grape tree that can produce grape-like berries that are rich in water-soluble ellagitannins and anthocyanins in fruit peel [9]. The berries are commonly called jaboticaba, which will turn dark red or purplish black when ripened. It has whitist and juicy pulp, and its taste is sweet and slightly sub-acid [10]. The shape of berries is round with the diameter appropriately 1 to 4 cm. Many products derived from the berries are available in the market, mainly because of high nutritional content and perishable structure. Natural vinegar is one of the potential jaboticaba products as it contains water-soluble anthocyanins that is well known for its antioxidant and anti-inflammatory properties [11]. Previously, spontaneous fermentation was performed by researchers from Brazil and they successfully produced jaboticaba vinegar with the concentration of acetic acid about 3% [12]. A year later, Dias et al. [13] reported the use of yeast (*Saccharomyces cerevisiae*) and immobilized mixed bacterial cultures of *Acetobacter aceti* and *Gluconobacter oxydans* to produce jaboticaba vinegar from the fruit surplus. Symbiotic growth of the mixed cultures is crucial as the metabolites that are produced during the first stage of ethanol production by yeasts must not inhibit the growth of acetic acid bacteria in the second stage of fermentation [14]. The metabolites produced during vinegar fermentation are unique compounds contributing to the flavor and aroma of fruit vinegar and possibly its medical values [15]. 

The present study aimed to determine the drying kinetic of perishable jaboticaba berries. The berries were also pretreated with 70% sugar and 10% salt solutions to accelerate the drying process. The dried berries were then used to prepare vinegar, and its quality was compared with the fresh berry vinegar from natural fermentation. The comparison was carried out based on the volatile and non-volatile metabolites using high-throughput hyphenated analytical techniques of headspace GC-MS and UHPLC-TWIMS-QTOFMS. Subsequently, the functionality of vinegar samples was also characterized for the antioxidant and antimicrobial capacity against pathogenic microbes.

## 2. Materials and Methods

### 2.1. Vacuum Oven Drying of Jaboticaba Berries

Ripen jaboticaba berries were used in the present study. They were pretreated by macerating them in the solution of 70% sugar and 10% salt overnight individually in two separate 1 L beakers. Each beaker contained about 200 g berries in 500 mL pretreatment solutions. A glass plate was put on top of the pretreatment solutions to prevent the berries from floating. After pretreatment, the berries were recovered, rinsed with distilled water and gently wiped off the surface moisture with tissue paper. They put evenly into squared weighing boats (8.5W × 8.5L × 2.5H cm) and then dried in a vacuum oven (Taisite, VO 52D, 41.5W × 37L × 34H cm, New York, NY, USA) which was operated at 40 °C and 0 MPa. Each weighing boat contained about 55 g of jaboticaba berries. Similarly, jaboticaba berries without pretreatment were also dried in the vacuum oven. Moisture saturated vapor was sucked out from the oven at day intervals. The drying process was carried out until the berries achieved constant weight.

### 2.2. Thin-Layer Drying Kinetic of Jaboticaba Berries

The mass loss of jaboticaba berries was calculated as moisture ratio (Equation (1)) and fitted to 5 thin-layer drying kinetic models: Newton, Handerson and Pabis, logarithmic, two term, Page, and Midilli et al. The goodness of the fit was examined based on the statistical parameters such as the correlation of coefficient (R^2^), adjusted correlation of coefficient (adjusted R^2^), root mean square error (RMSE), and chi-squared value (χ^2^), as shown in Equations (2)–(5).

(1) $$\mathrm{MR}=\frac{M-M_{e}}{M_{o}-M_{e}}$$

(2) $$R^{2}=1-\left[\frac{\sum_{i=1}^{N}{\left(MR_{\exp}-MR_{\mathrm{pre}}\right)}^{2}}{\sum_{i=1}^{N}{\left(MR_{\exp}-MR_{\exp,mean}\right)}^{2}}\right]$$

(3) $$\mathrm{Adjusted}\;R^{2}=1-\frac{\left(1-R^{2}\right)\left(N-1\right)}{\left(N-v-1\right)}$$

(4) RMSE=[1N∑i=1N(MRexp−MRpre)2]12

(5) $$\chi^{2}=\sum_{i=1}^{n}\left(\frac{MR_{exp}{}_{}-{\mathrm{MR}}_{\mathrm{pre}}}{{\mathrm{MR}}_{\mathrm{pre}}}\right)$$

where M is the moisture content, M_o_ is the initial moisture content and M_e_ is the equilibrium moisture content. The constant n is the number of experiment, whereas v is the number of independent variable in the regression analysis. The value of R^2^ is close to unity, but the value of RSME and χ^2^ are close to zero represent a suitable agreement between the experimental results (exp) and model predicted data (pre).

The effective moisture diffusivity (D_eff_) of jaboticaba berries can be also determined using Fick’s law of diffusion. Equation (6) can be applied to explain the drying behavior of a spherical geometry of berries for a long period of drying time. The equation can then be converted into a linear equation as shown in Equation (7). The slope of the linear line is used to determine the D_eff_ of jaboticaba berries in a vacuum oven dryer.

(6) $$\frac{6}{\pi^{2}}\overset{\infty }{\underset{n=0}{\sum }}\frac{1}{n^{2}}\exp(\frac{-n^{2}\pi^{2}D_{eff}t}{r^{2}})$$

(7) $$\ln\left(\mathrm{MR}\right)=\ln\left(\frac{6}{\pi^{2}}\right)-\left(\frac{n^{2}D_{eff}}{r^{2}}\right)t$$

where r is the radius of the jaboticaba berry and t is the drying time

### 2.3. Natural Fermentation of Jaboticaba Vinegar

The fresh (300 g) and dried (non-treated, 70 g) berries of jaboticaba were put into individual sterile Schott bottles and topped up with drinking water (600 mL) to cover the berries. The sample bottles were covered with muslin cloth and left aside for fermentation in a well-ventilated area. The sample bottles were shaken manually every day during the first stage of alcoholic fermentation. The supernatant was collected on day 7 and transferred to another cleaned Schott bottle for the second stage of acetic acid fermentation. Similarly, the bottles were covered with muslin cloth and intermittently shaken for 3 months.

### 2.4. Physiochemical Analysis of Jaboticaba Vinegar

The pH and conductivity of vinegar samples were measured using a pH meter (LAQUAtwin-pH-11, Horiba Scientific, Kyoto, Japan) and a conductivity meter (LAQUAtwin-EC-11, Horiba Scientific, Japan), respectively. The pH buffer solutions at pH 4.01 (Model 514-4) and 7.00 (Model 514-7) were used to calibrate the pH meter, whereas the conductivity solution at 1.413 mS/cm (Model514-22) was used to calibrate the conductivity meter before use. The sugar content of vinegar samples was measured using a brix refractometer (MA871, Milwaukee Instruments, Milwaukee, WI, USA).

The acidity of vinegar samples was determined using the titrimetric method (Metrohm 665 Dosimat autotitrator, Herisau, Switzerland). A 10 g sample was weighted in a beaker, and 600 mL of distilled water was added into the beaker with two drops of phenolphthalein as indicator. The solution was titrated with 0.1 M sodium hydroxide until the solution turned stable pink. Equation (8) is used to calculate the acidity of vinegar expressed in acetic acid percentage.

(8) $$\mathrm{Acidity}\;=\frac{\left(\mathrm{Titrant}\;\mathrm{volume}\;\left(\mathrm{mL}\right)\times \;\mathrm{NaOH}\;\left(M\right)\times \;\mathrm{Molar}\;\mathrm{mass}\;\mathrm{of}\;\mathrm{acetic}\;\mathrm{acid}\;\left(g/\mathrm{mol}\right)\right)}{\mathrm{Sample}\;\mathrm{weight}\;\left(\mathrm{mg}\right)}\times 100$$

The color of jaboticaba vinegar samples was spectrophotometrically measured at 4 different wavelengths, namely 430 nm, 520 nm, 580 nm, and 620 nm [16]. Citrate-phosphate or McIlvaine buffer at pH 3.0 was used to dilute vinegar samples to prevent the color change of pH-sensitive compounds. This citrate-phosphate buffer was prepared by mixing 4.11 mL of disodium hydroxyphosphate (0.2 M) and 15.89 mL of citric acid solution (0.1 M). The change of absorbance at different concentrations of vinegar samples was plotted. The slope of the linear curve is expressed as the absorption coefficient (Equation (9)).

(9) $$E\;=\frac{A}{C.l}$$

where ε is the absorption coefficient, A is the absorbance, C is the sample concentration, and l is the length of the cuvette (1 cm).

The ratio of the absorbance at different wavelengths was applied to determine brown and blue indexes as presented in Equations (10) and (11). Fresh berries were also macerated in water in a ratio of 1:1 for 24 h. The solution was then harvested and used as a positive control.

Brown Index = A_430_/A_520_ (10)

Blue Index = A_580_/A_520_ (11)

### 2.5. Phenolic Composition of Vinegar

The phenolic composition of samples was colorimetrically assayed for total phenolic content (TPC), total flavonoid content (TFC), and total anthocyanin content (TAC) using a UV-Vis spectrophotometer (Shimadzu UV-1800, Kyoto, Japan).

TPC was determined using Folin–Ciocalteu reagent according to the procedures reported by Alezandro et al. [17]. A 0.5 mL sample, 0.3 mL Folin–Ciocalteu reagent, and 2 mL sodium carbonate (15% *w*/*v*) were sequentially pipetted into a 5 mL centrifugal tube. The mixture was then topped up with distilled water (2.2 mL) to make up the total volume of the mixture into 5 mL. The mixture was incubated for 2 h at room temperature in a dark place. Its absorbance was measured using the spectrophotometer at 798 nm. Gallic acid (Sigma-Aldrich, Burlington, NJ, USA) was used as the standard chemical. The results are expressed as mg gallic acid equivalents (GA) per gram sample. The equation of the calculation is presented in Equation (12).

(12) $$\mathrm{Total}\;\mathrm{Phenolic}\;\mathrm{Content}\;\left(\mathrm{TPC}\right)\;\mathrm{or}\;\mathrm{Total}\;\mathrm{Flavonoid}\;\mathrm{Content}\;\left(\mathrm{TFC}\right)=\frac{C.V}{m}$$

C: concentration of gallic acid or quercetin from the calibration curve 

m: weight of propolis extract (g) 

V: volume of propolis extract (mL)

TFC was measured using 5% aluminum chloride according to the procedures reported by Kung et al. [18]. A 2 mL sample and 3 mL of aluminum chloride were pipetted into a 5 mL centrifugal tube. The solution was well mixed and incubated in a dark place for 30 min prior to the absorbance measurement at 510 nm using the spectrophotometer. Equation (12) was used to calculate TFC using quercetin as a standard chemical. The result is expressed as mg quercetin equivalent (Q) per gram sample.

TAC was determined using the pH differential method [19]. Two types of buffer solution at different pH values were prepared in this assay. The aqueous buffer (1 L) was prepared by dissolving KCl (1.86 g) in distilled water and adjusting the pH to 1.0 ± 0.05 using HCl (0.01 M). The acetate buffer (1 L) was prepared by dissolving sodium acetate (54.43 g) in distilled water and adjusting the pH to 4.5 ± 0.05 using HCl. One portion of the sample was added into four portions of aqueous buffer at pH 1 and acetate buffer at pH 4.5 each. The absorbance was measured at 520 nm and 700 nm after 30 min of incubation. Cyanidin-3-glucoside was used as a positive control. Equation (13) was used to calculate TAC. The results are expressed in milligram cyanidin-3-glucoside equivalent (C3G) per gram sample.

(13) $$\mathrm{Total}\;\mathrm{anthocyanin}\;\mathrm{content}\;\left(\mathrm{TAC}\right)=\frac{A\;\times \;\mathrm{MW}\;\times 1000}{\varepsilon \;\times \;p\;\times \;s}$$

where 

A: absorbance, A = $\left(A_{520\;}-{\;A}_{700}\right)\mathrm{pH}1.0-\left(A_{520\;}-{\;A}_{700}\right)\mathrm{pH}4.5$

MW: molecular weight of cyanidin-3-glucoside (449.2 g/mol)

ε: extinction coefficient (26,900 L/(mol.cm))

p: pathlength (1 cm)

s: sample concentration (g/L)

### 2.6. Antioxidant Capacity of Vinegar

The antioxidant capacity of vinegar samples was compared using different colorimetric assays that were performed based on the capability of samples acting as either a free radical scavenger, radical cation scavenger, or reducing agent. The scavenging activity of samples was determined using DPPH (2,2-diphenyl-1-picrylhydrazyl), ABTS (2,2′-azino-bis(3-ethylbenzothiazoline-6-sulfonic acid)), and 2,2′-azobis (2-methyl-propanimidamide) dihydrochloride (AAPH) reagents to generate free radicals, radical cations, and oxygen radicals, respectively. The reducing power of samples was measured based on the reduction in colorless FeIII-TPTZ (ferric-tripyridyltriazine) into blue Fe(II)-TPTZ.

The DPPH assay was carried out according to the procedures reported by Alezandro et al. (2013). A 2 mL sample and 2 mL methanolic DPPH (0.1 mM) were mixed thoroughly and then incubated in a dark room for 30 min prior to the absorbance measurement at 517 nm of the spectrophotometer. The purple-colored DPPH radicals were changed to a yellowish solution after being scavenged by antioxidative compounds in samples. The inhibitory percentage of DPPH free radicals can be calculated using Equation (14). The results are expressed in the effective concentration of the sample to exhibit 50% inhibition (IC50).

(14) $$\mathrm{Radical}\;\mathrm{inhibition}\;\left(\%\right)=\frac{A_{o}-A_{1}}{A_{o}}\times 100$$

A_0_ is the absorbance of the control, and A_1_ is the absorbance of the sample.

The ABTS assay was performed according to the procedures reported by Chew et al. [20]. An equal volume of ABTS (7 mM) and potassium persulfate (2.45 mM) was added and incubated overnight in a dark place to generate radical cations. The solution was diluted with distilled water until its absorbance achieved the value of 1.00 at 734 nm. The diluted ABTS solution (2 mL) was added into different concentrations of sample solution (100 mL). The intensity of the blue-colored solution was getting lighter or even colorless after being scavenged by antioxidative compounds in samples for 6 min in a subdued light condition. The absorbance of the sample was measured again at 734 nm using the spectrophotometer.

The ORAC (oxygen radical absorbance capacity) assay was carried out to determine the total antioxidant capacity of samples according to the procedures described by Andrade et al. [21] with modification. A 1.50 mL fluorescein working solution (6.30 mmol/L) and 0.75 mL samples (1–3 mg/mL) or standard Trolox (2.0–10.0 mg/L) were mixed and incubated at 37 °C for 15 min. Then, 0.75 mL AAPH working solution (153 mmol/L) was added to the mixture. The absorbance was recorded every 90 s at 520 nm using a spectrophotometer for 1 h. The ORAC value was calculated by subtracting the reaction curve area of the blank from the reaction curve area of the sample. The reaction curve area was plotted based on the decreasing absorbance over the reaction time for 1 h. The results are expressed in micromole Trolox equivalent (T) per g sample.

The FRAP assay was determined according to the procedures reported by Chew et al. [20]. FRAP solution was freshly prepared by adding 300 mM acetate buffer (25 mL) at pH 3.6, 10 mM TPTZ (2.5 mL) in HCl (40 mL), and 20 mM ferric chloride (2.5 mL). An equal volume of FRAP solution (1 mL) was mixed with different concentrations of the sample (1 mL), and the absorbance of the mixture was read at 593 nm. Trolox (0.004–2.000 mg/mL) was used as the standard chemical. The reducing power is evaluated using Equation (15).

(15) $$\mathrm{Ferric}\;\mathrm{reducing}\;\mathrm{antioxidant}\;\mathrm{power}\;\left(\%\right)=\frac{A_{1}-A_{o}}{A_{s}-A_{o}}\times 100$$

A_o_ is the absorbance of the control, A_S_ is the absorbance of ascorbic acid, and A_1_ is the absorbance of the sample.

### 2.7. Antimicrobial Capacity of Vinegar

Five pathogenic microbes, such as *Escherichia coli* (ATCC 8739), *Pseudomonas aeruginosa* (ATCC 9027), *Salmonella enterica* subsp. *enterica* serovar Typhimurium (ATCC 14028), *Staphylococcus aureus,* and *Listeria monocytogenes* (ATCC 19115), were used to examine the antimicrobial capacity of vinegar samples using the microtiter broth dilution method. A few isolated colonies from an overnight culture of brain heart infusion agar were transferred into phosphate buffered saline (PBS) and standardized to McFarland 0.5 turbidity in order to obtain a microbial density equivalent of about 1.5 × 10^8^ CFU/mL. The suspension (2 mL) was then added to 40 mL PBS in a ratio of 1:20 inoculum. The inoculum was also streaked on blood agar to confirm the purity of the suspension. A total of 100 µL of brain heart infusion broth (BHIB) was placed into wells of a microtiter plate. Another 100 µL of vinegar samples was added and mixed before serial dilution was performed in each well. Consequently, 10 µL of the inoculum was added into each well and incubated at 35 °C for 20 h. The absorbance of each well was measured using a microplate reader (Lonza KQCL, ELx808LBS, Basel, Switzerland) at 630 nm. The minimum inhibition concentration of vinegar samples was determined from the well with the complete inhibition of microbial growth preceding the one with a significant increment in absorbance value. BHIB with inoculum and sample blank was prepared as a control in this experiment.

### 2.8. Headspace GC-MS

A hyphenated analytical tool of headspace (Agilent 7697A, CA, USA) coupled with gas chromatography-mass spectrometry was used to identify volatile compounds released from the heated vinegar samples (15 mL) in 20 mL screw-cap vials. The sample vials were sealed and thermally heated at 80 °C for 30 min in a closed system. The temperature of the loop and transfer line in the headspace were maintained at 90 °C and 110 °C, respectively. A gas chromatography (Agilent 8890) integrated with a mass analyzer (Agilent 5977 MSD) was then used to analyze the released compounds. The temperature of the injection port was set at 220 °C and configured for split mode at the ratio of 10:1. The oven temperature was started at 40 °C and held for 5 min, increased to 230 °C at 10 °C/min, maintained at 230 °C for 10 min, and heated to 260 °C. The scan range of *m*/*z* was 30–350 amu. Helium was used as the carrier gas at 1 mL/min. A nonpolar column (Agilent J&W DB-624) with the dimension of 0.25 mm ID × 60 m L × 1.4 µm thickness was used for compound separation. The ion source of the mass detector was the electronic impact (70 eV) with a source temperature of 230 °C and quadrupole temperature of 150 °C. The other setting parameters were solvent delay, 4.4 min; stop time, 10 min; and scan speed, 1562.

### 2.9. UHPLC-TWIMS-QTOFMS

An ultra-high performance liquid chromatography (UHPLC) of ACQUITY UPLC I-Class system was coupled with a Vion TWIMS-QTOF hybrid mass spectrometer and a Lock Spray ion source (Waters, Milford, CT, USA) to perform metabolite screening. Compounds were chromatographically separated using a column ACQUITY UPLC HSS T3 (100 mm × 2.1 mm × 1.8 μm), which was maintained at 40 °C. The mobile phase consisted of water (0.1% formic acid) and acetonitrile. Its gradient profile is as follows: 0–0.5 min, 1% B; 0.5–16.0 min, 1–35% B; 16.0–18.0 min, 35–100% B; 18.0–20.0 min, 1% B. The flow rate was set at 0.6 mL/min, and the injection volume was 1 μL.

The ion source was operated at the positive and negative mode under the following specific conditions; capillary voltage, 1.50 kV; reference capillary voltage, 3.00 kV; source temperature, 120 °C; desolvation gas temperature, 550 °C; desolvation gas flow, 800 L/h, and cone gas flow, 50 L/h. Nitrogen (>99.5%) was employed as desolvation gas and cone gas. Argon (99.999%) was used as collision-induced dissociation (CID) gas. Data were acquired in high-definition MSE (HDMSE) mode in the range *m*/*z* 50–1500 at 0.1 s/scan. Two independent scans were fixed low collision energy at 4 eV, and high collision energy ramped from 10 to 40 eV.

## 3. Results and Discussion

### 3.1. Effects of Osmotic Dehydration Pretreatment

Jaboticaba berries were vacuum-dried in an oven at 40 °C. Some berries were subject to pretreatment using 70% sugar and 10% salt solutions overnight prior to drying. Consequently, the weight of the berries was monitored until a constant weight was achieved. Table 1 shows that berries that were pretreated with 70% sugar solution achieved the highest mass loss, which is about 13.2% more than berries without any pretreatment. However, 10% salt solution pretreatment showed 8.2% mass gain and the lowest total mass loss after vacuum drying. A small portion of salt diffused into berries resulted from osmotic pressure during pretreatment. The existence of salt in berries increased water-holding capacity, and therefore, berries pretreated with salt were found to have a lower total mass loss.

**Figure 1:**
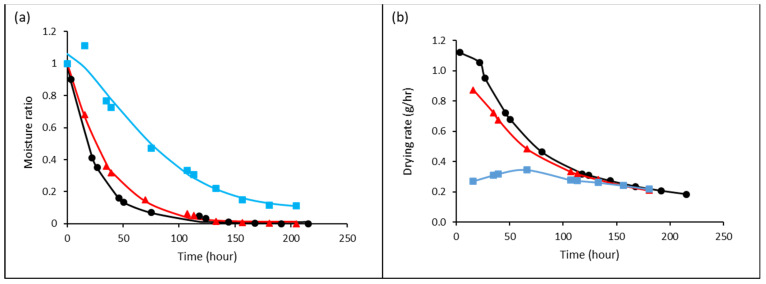
Thin-layer drying kinetic model (line) is well fitted to the experimental data of the moisture ratio of jaboticaba berries pretreated with 70% sugar (▲) and 10% salt (■) compared to berries without any pretreatment (●). (**a**) The degradation of moisture ratio with drying time and (**b**) The drying rate of jaboticaba berries with time.

Jaboticaba berries are perishable and deformable because of their high moisture content (>80%). Hence, drying would cause shrinkage simultaneously during moisture diffusion. The stress condition led to the collapse of cellular structure, geometrical change, and capillary contraction, thus affecting the moisture diffusivity of jaboticaba berries [22]. The drying rate of jaboticaba berries decreasing with drying time without a constant rate period was observed (Figure 1). Only two falling rate periods were observed to explain the movement of liquid water to the surface of berries and the evaporation of surface moisture to the surrounding. Diffusion is the dominant mechanism of water removal from jaboticaba berries in a vacuum dryer at 40 °C. The resistance of internal moisture diffusion controlled the overall performance of drying.

However, salt-pretreated berries showed to have different drying behavior. The drying rate of salt-pretreated berries was initially increased for the first 60 h and then slowly decreased until mass equilibrium was reached. The initial increment of drying rate was most probably due to the fast heating process in the presence of salt to achieve thermal equilibrium between the fruit surface and surrounding air, and therefore, surface moisture could be vaporized rapidly [23]. As the drying continued, the critical moisture content of jaboticaba berries would be achieved, and subsequently, moisture diffusion was the dominant drying mechanism, as explained by the falling rate period in Figure 1b. 

### 3.2. Thin-Layer Drying Kinetic of Jaboticaba Berries

A few thin-layer drying kinetic equations were fitted to the experimental data of drying jaboticaba berries in a vacuum oven. The kinetic constant (k), drying constants (a and b), correlation of coefficients (R^2^), adjusted correlation of coefficients (adjusted R^2^), root mean squared error (RMSE), and chi (χ^2^) values of each model are presented in Table 2. The drying behavior of jaboticaba berries was found to follow thin-layer kinetics with high R^2^ > 0.99, low RMSE < 0.032, and χ^2^ < 0.032. Based on the data, Midilli et al. model is the most suitable thin-layer model to explain the drying behavior of jaboticaba berries in a vacuum oven at 40 °C. The selection is based on the high R^2^ and adjusted R^2^, low RMSE, and χ^2^ values, as well as the kinetic and drying constants, which are non-zero. R^2^ tends to increase as the number of effects is included in the model equation. The decrease in adjusted R^2^ is < 0.02, and the small difference explains that the increase in effects does not overestimate the goodness of the fit. The model fits well with the drying behavior of jaboticaba berries. Even the berries had been pretreated with 70% sugar and 10% salt solutions. The recent works of Rasooli Sharabiani et al. [24] also reported that Midilli et al. model could well explain the drying behavior of apple slides under convective drying and microwave drying.

Fick’s second law was used to calculate the effective moisture diffusivity. The moisture diffusion coefficient ranged from 6.73 × 10^−^^11^ to 2.52 × 10^−^^10^ m^2^/s, which was lower than the values reported for seedless grapes that dried in a microwave vacuum dryer [25]. The lower moisture diffusivity could be partly due to the thicker fruit peel that limited the diffusion of moisture. Somehow, the effective moisture diffusivity differs depending on fruit composition, microstructure, and drying variables. Moisture migration during drying is a very complex process and often involves more than one transport mechanism, such as molecular diffusion, vapor diffusion, surface diffusion, and hydrostatic pressure differences [26]. This complex mass transfer phenomenon can be explained by using effective moisture diffusivity to describe all parameters influencing the drying rate.

In the present study, non-treated berries were shown to have higher effective moisture diffusivity (2.52 × 10^−^^10^ m^2^/s) than the values of 70% sugar (1.84 × 10^−^^10^ m^2^/s) and 10% salt (6.73 × 10^−^^11^ m^2^/s)-pretreated berries. The pretreated berries had changed their fruit composition resulting from osmotic pressure, therefore, causing lower moisture diffusivity. The non-treated and sugar-pretreated berries could achieve almost similar mass equilibrium after 130 h of drying. However, salt-pretreated berries required a longer time, 180 h, to achieve constant weight. The observation explains that salt-pretreated berries have a higher water-holding capacity.

### 3.3. Physiochemical Properties of Jaboticaba Vinegar

The physiochemical properties of vinegar samples were analyzed for pH, conductivity, acidity, and color index. Table 3 shows that both vinegars have almost similar pH and acetic acid content but higher conductivity for dried fruit vinegar. Most probably, more dissolved solid and metal elements from dried berries diffuse into the aqueous phase and contribute to higher conductivity. The destruction of fruit cells resulting from shrinkage during drying eased the diffusion of dissolved solids during vinegar fermentation.

**Figure 2:**
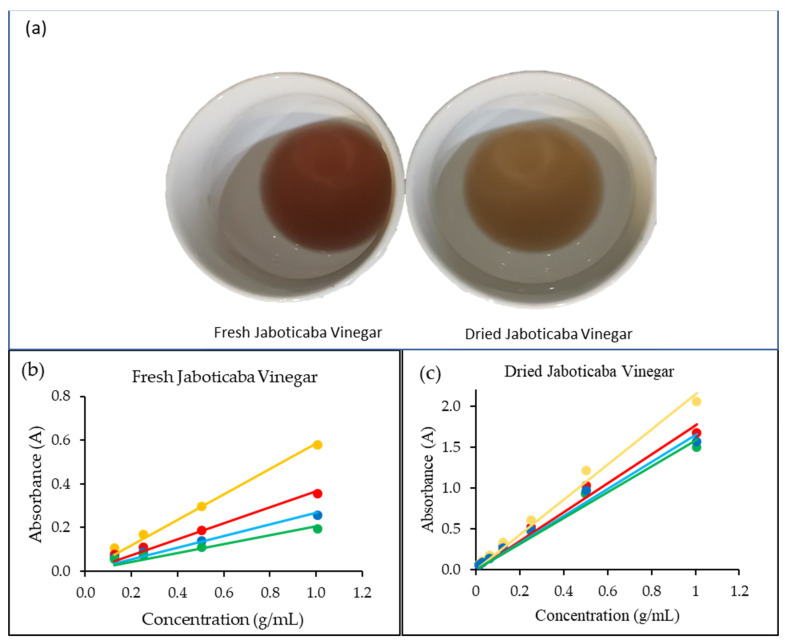
(**a**) Picture of jaboticaba vinegar fermented from fresh and dried berries, (**b**) absorbance of fresh jaboticaba vinegar at different concentrations and different wavelengths, and (**c**) absorbance of dried jaboticaba vinegar at different concentrations and different wavelengths. Yellow line denotes 430 nm, red line denotes 520 nm, blue line denotes 520 nm, and green line denotes 620 nm.

The color of vinegar would affect the taste perception of consumers. It was also analyzed spectrophotometrically using the brown and blue indexes in the visible region of the spectrum. The indexes are usually used to measure the color change of juices after juice processing. Color measurement can be used to determine the extent of oxidative browning during vinegar fermentation. The brown index is suitable for a yellowish solution, whereas the blue index is more appropriate for a purplish solution. It is important to use citrate-phosphate buffer for sample dilution as anthocyanins in vinegar are strongly pH dependent. Table 3 shows that the color indexes of fresh berry vinegar do not change significantly compared to the fresh berry extract solution from maceration as a positive control in the present study. Anthocyanins in fresh berry vinegar appeared to be stable against oxidation during vinegar production. However, vinegar prepared from dried berries showed a reduction in the brown index and an increment in the blue index. Visual inspection clearly observes that dried berry vinegar is more brownish, as presented in Figure 2a. The observation could be explained by some extent of oxidation to the thermal-sensitive compounds in berries resulting from drying. The recent works of Ndiaye et al. [27] reported that both brown and blue indexes of Hibiscus sabdariffa juice were increased with the increase in temperature and storage duration.

The absorption coefficient (curve slope) of dried berry vinegar was higher than fresh berry vinegar at different wavelengths (Figure 2). The increment of absorbance values explained the degradation of compound stability due to oxidation during fruit drying. The degradation might produce metabolites visible at that particular wavelength. Of the wavelengths, both vinegar samples also showed to have the highest absorbance at 430 nm, followed by 520 nm, 580 nm, and 620 nm. Since the vinegar samples have the highest absorptivity at 430 and 520 nm, they are apparently yellowish-red in color, as seen in Figure 2a.

### 3.4. Phytochemical Content and Antioxidant Capacity

The vinegar samples were analyzed for their proximate phytochemical components spectrophotometrically. The results found that fresh berry vinegar had higher flavonoids and anthocyanins, which was attributed to higher scavenging activity against free (DPPH) and peroxyl (ORAC) radicals than dried berry vinegar (Table 4). Lower IC50 DPPH explained that a lower concentration of vinegar samples was required to achieve 50% inhibition. Flavonoids, including water-soluble anthocyanins, were unlikely to increase the reducing power (FRAP) and scavenge radical cations (ABTS). On the other hand, dried berries could produce vinegar with higher phenolic content. The phenolics of jaboticaba berry vinegar appeared to be a suitable cationic radical scavenger because a comparable performance of IC50 ABTS capacity was observed for both fresh and dried berry vinegar samples. Most probably, the phenolics were attributed to the products of bond breaking from tannins and lignins in fruit peel. Apparently, cell collapse resulting from the drying process could assist the hydrolysis of tannins and lignins under acidic conditions during vinegar production.

Owing to the strong and sharp pungent smell, the volatile organic compounds of vinegar samples were analyzed using headspace GC-MS. The volatile organic compounds were mostly composed of acetic acid, namely 88.5% from fresh fruit vinegar and 64.3% from dried fruit vinegar. The smell of fresh berry vinegar was sharper than dried berry vinegar. This included a strong cheesy and sweaty smell that was contributed by 2- and 3-methylbutanoic acids in fresh berry vinegar. It was found that higher methanol content (4.1%) was also detected in dried fruit vinegar. Only 0.6% of methanol was detected from the vapor of fresh fruit vinegar vapor. Bourgeois et al. [28] reported that methanol could be produced from pectin, which was hydrolyzed partially to pectic acid and methanol by pectinesterase. The dried fruit vinegar also contained acetoin (3-hydroxy-2-butanone), which is a pale to yellowish liquid with a pleasant yogurt creamy odor and a fatty butter taste. It is a bacteria- and yeast-fermented product, contributing to natural flavor and aroma [29].

The non-volatile compounds were then identified using the high sensitivity and mass accuracy of UHPLC-TWIMS-QTOFMS. Different classes of compounds, such as nitrogen-containing compounds, including alkaloids and amino acid derivatives; phenolics, including phenolic acid, tannins, and lignins; terpenoids and saponins; and sugars and organic acids were detected from the vinegar samples from non-targeted mass screening. Compounds under the class of organic acids, flavonoids, phenolics, and their derivatives are sorted, and only those with theoretical fragments >2 and mass error <10 mDa are listed in Table S1. The results found that more compounds were detected in dried berry vinegar from both positive and negative ion modes than those detected in fresh berry vinegar. Possibly, berry metabolites could be easier to diffuse into an aqueous medium from structurally disturbed cells as a result of drying. The detected organic acids were citric acid, quinic acid, shikimic acid, and chlorogenic acid. Vacuum drying at low temperatures appeared to degrade 28–77% anthocyanins based on their peak intensities in vinegar samples. The vinegar samples contained a wide range of anthocyanins, such as hibiscetin-3-O-glucoside, peonidin 3-glucoside, pelargonidin 3-glucoside, cyanidin, delphin, and malvidin 3,5-diglucoside (Table S1). This is the first time hibiscetin-3-O-glucoside reported in jaboticaba. Pelargonidin 3-glucoside was found to be the least stable anthocyanin because it was detected in fresh berry vinegar but could not be detected in dried berry vinegar. Pelargonidin 3-glucoside was one of the anthocyanins that contributed to the red-colored vinegar from fresh berries. The vinegar samples were also rich in glycosylated flavonoids, especially under the classes of flavone, isoflavone, and xanthone. The compounds have been reported to possess remarkable antioxidant and anti-inflammatory properties from naturally fermented fruit vinegar [30].

**Figure 3:**
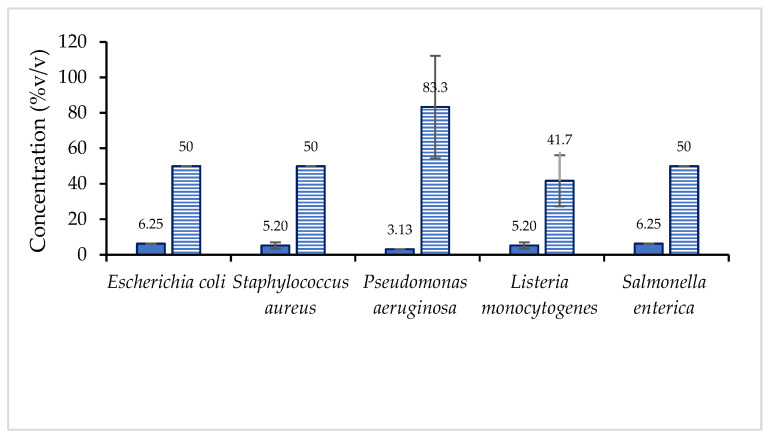
Minimum inhibition concentration of vinegar samples prepared from fresh (solid bar) and dried (line bar) jaboticaba berries.

The biological activity of vinegar samples was also examined for their performance in inhibiting the growth of pathogenic microorganisms such as *E. coli, S. enterica, P. aeruginosa, S. aureus,* and *L. monocytogenes* using the method of microtiter broth dilution. The performance of fresh berry vinegar was better because it required a lower minimum concentration to inhibit the growth of the microbes than the concentration required by dried fruit vinegar (Figure 3). The fresh berry vinegar was most effective in inhibiting the growth of *P. aeruginosa,* but its counterpart, dried berry vinegar, showed the poorest performance against *P. aeruginosa*. Therefore, drying would diminish the quality of vinegar, especially its biological function against the growth of pathogenic microbes. Most probably, the inhibition was contributed by flavonoids, particularly water-soluble anthocyanins, because they were more likely in the fresh berry vinegar. The remarkable antimicrobial property of natural vinegar has also been reported in many studies [31,32,33].

## 4. Conclusions

The low temperature of vacuum drying jaboticaba berries was found to follow the thin-layer kinetic model of Midilli et al. satisfactorily with high r^2^ > 0.97, low RMSE < 0.06, and χ^2^ < 0.007, even the berries were pretreated with 70% sugar and 10% salt solutions. The pretreatment most probably changed the chemical composition of the berries due to the effect of osmotic pressure, thus causing the effective moisture diffusivity to be lower than the non-treated berries. Moisture diffusion was the dominant mechanism controlling the drying behavior of jaboticaba berries. Natural fermentation successfully produced jaboticaba vinegar from both fresh and dried berries with almost similar pH 3 and acidity expressed in 3% acetic acid. Vacuum oven drying showed to have some extent of oxidation onto labile anthocyanins, which can be seen from the color change. Different volatile and non-volatile compounds were also detected using high-sensitivity hyphenated analytical tools. Preparing jaboticaba vinegar from fresh berries would enhance its performance to inhibit pathogenic microbes, in addition to being a suitable radical scavenger. Therefore, natural fruit vinegar from fresh jaboticaba berries offers high antioxidant capacity due to its flavonoids, especially water-soluble anthocyanins.

## Figures and Tables

**Table 1:** Mass loss of jaboticaba berries pretreated with 70% sugar and 10% salt solutions.

Pretreatment	Mass Loss by Pretreatment (%)	Total Mass Loss (%)
Non-pretreatment	0	76.6 ± 1.1
70% sugar solution	26.8 ± 0.6	89.8 ± 1.3
10% salt solution	8.2 ± 1.1 *	61.4 ± 1.2

* mass gain.

**Table 2:** Six thin-layer drying kinetic models and their constants for the goodness of fit.

Thin-Layer Drying Model	Without Pretreatment					Pretreatment with 70% Sugar Solution					Pretreatment with 10% Salt Solution				
	Constant	R^2^	Adjusted R^2^	RMSE	χ^2^	Constant	R^2^	Adjusted R^2^	RMSE	χ^2^	Constant	R^2^	Adjusted R^2^	RMSE	χ^2^
Newton	k = 0.0391	0.9978	0.9978	0.0159	0.001	k = 0.0277	0.9976	0.9976	0.016	0.0010	k = 0.0195	0.9478	0.9478	0.0100	0.0179
MR = exp(−kt)															
Handerson and Pabis	k = 0.0397	0.9980	0.9978	0.0152	0.0009	k = 0.0281	0.9978	0.9974	0.0155	0.0010	k = 0.0114	0.9619	0.9565	0.0707	0.0102
MR = a.exp(−kt)	a = 1.0126					a = 1.0117					a = 1.1328				
Logarithmic	k = 0.0408	0.9985	0.9981	0.0132	0.0007	k = 0.0281	0.9978	0.997	0.0155	0.0010	k = 0.0114	0.9619	0.9492	0.0707	0.0102
MR= a.exp(−kt) + b	a = 1.0034					a = 1.0117					a = 1.1328				
	b = 0.0111					b = 0					b = 0				
Two term	k_1_ = 0.0397	0.9979	0.9975	0.0152	0.0009	k_1_ = 0.0281	0.9978	0.997	0.0155	0.0010	k_1_ = 0.0114	0.9619	0.939	0.0707	0.0102
a.exp(-k_1_t) + b.exp (-k_2_t)	k_2_ = 0.0397					k_2_ = 1.3437					k_2_ = 0				
	a = 0.5063					a = 1.0117					a = 1.1328				
	b = 0.5063					b = 0					b = 0				
Page	k = 0.0347	0.9979	0.9977	0.0155	0.001	k = 0.0214	0.9981	0.9978	0.0142	0.0008	k = 0.0013	0.97	0.9657	0.0627	0.0083
MR = exp(−kt^n^)	n = 1.0360					n = 1.0729					n = 1.4373				
Midilli et al.	k = 0.0355	0.9984	0.9978	0.0137	0.0007	k = 0.0192	0.9982	0.9972	0.0141	0.0007	k = 0.0018	0.9775	0.964	0.0543	0.0061
MR = a exp(−kt^n^) + bt	n = 1.0339					n = 1.1084					n = 1.4121				
	a = 1.0088					a = 1.0044					a = 1.0623				
	b = 0.00006					b = 0.00006					b = 0.0004				

**Table 3:** Physiochemical properties of jaboticaba vinegar from fresh and dried berries.

Parameter	Maceration Solution	Fresh Fruit Vinegar	Dried Fruit Vinegar
pH	4.5 ± 0.1	3.0 ± 0.1	3.1 ± 0.1
Conductivity (mS/cm) *	2.35 ± 0.30	3.15 ± 0.25	4.25 ± 0.33
Acidity (% acetic acid)	0	3.1 ± 0.1	3.0 ± 0.1
Brown index *	1.762 ± 0.037	1.794 ± 0.025	1.170 ± 0.030
Blue index *	0.691 ± 0.073	0.668 ± 0.012	0.937 ± 0.003

* There is a significant difference between the values contributed by fresh and dried berry vinegar samples using a *t*-test paired with two samples for mean.

**Table 4:** Phytochemical content and antioxidant capacity of jaboticaba vinegar fermented from fresh and dried berries.

Parameter	Fresh Berry Vinegar	Dried Berry Vinegar
Solid (%)	1.8 ± 0.2	1.6 ± 0.1
Sugar (°Bx)	3.0 ± 0.1	1.1 ± 0.2
Total phenolic content (mg GA/g) *	39.49 ± 0.48	54.71 ± 3.16
Total flavonoid content (mg Q/g) *	6.49 ± 0.13	1.91 ± 0.15
Total anthocyanin content (mg C3G/g) *	2.71 ± 0.37	0.24 ± 0.13
IC50 DPPH (Trolox, 0.005 mg/mL) *	0.94 ± 0.23	3.34 ± 0.19
IC50 ABTS (Trolox, 0.114 mg/mL)	1.59 ± 0.29	1.47 ± 0.40
ORAC (µmol T/g) *	26.93 ± 2.56	6.64 ± 1.29
FRAP (*f*.mol T/kg)	0.252 ± 0.063	0.205 ± 0.038

* There is a significant difference between the values contributed by fresh and dried berry vinegar samples using a *t*-test paired with two samples for mean.

## Data Availability

Data are included in the article and supplementary materials.

## Supplementary Materials

The following supporting information can be downloaded at: https://www.mdpi.com/article/10.3390/foods12010065/s1, Table S1: Organic acids, flavonoids, phenolics, tannins, lignins, and their derivatives that detected in fresh and dried berries vinegars at the positive and negative ion modes of UHPLC-TWIMS-QTOFMS.
