# Supplementary material for: Drying Kinetic of Jaboticaba Berries and Natural Fermentation for Anthocyanin-Rich Fruit Vinegar
Source: Foods. 2022 Dec 23;12(1):65. doi: 10.3390/foods12010065 (PMC9818337; doi:10.3390/foods12010065)
Supplement: Supplementary file 1 [file foods-12-00065-s001.zip › foods-2087469-supplementary.pdf]

Table S1. Organic acids, flavonoids, phenolics, tannins, lignins and their derivatives that detected in fresh and dried berries vinegars at the positive and negative ion modes of UHPLC-TWIMS-QTOFMS

Fresh berry vinegar (positive ion)			Dried berry vinegar (positive ion)		
**Compounds**	**Neutral mass (Da)**	**Observed m/z**	**Compounds**	**Neutral mass (Da)**	**Observed m/z**
**Organic acids & their derivatives**			**Organic acids & their derivatives**		
			Citric acid	192.027	215.0152
1,5-Dimethyl citrate	220.0583	243.0465	1,5-Dimethyl citrate	220.0583	243.0467
9,12-Dihydroxy-15-nonadecenoic acid	328.26136	351.2493	9,12-Dihydroxy-15-nonadecenoic acid	328.26136	351.2495
Propyl chlorogenate	396.14203	397.1488	Propyl chlorogenate	396.14203	397.1492
**Flavonoids & their derivatives**			**Flavonoids & their derivatives**		
			Astragaline A (kaempferol based flavonoid)	209.06881	210.0753
Astragaline C	253.09502	254.1034	Astragaline C (flavonoid)	253.09502	254.1017
			Astragaline E (flavonoid)	292.10592	331.0686
Quercetin_1	302.04265	303.0489			
Cyanidin	322.02442	345.0168	Cyanidin	322.02442	345.0174
Gardenin E	390.09508	391.1018			
			Sanggenon H (flavanone)	354.11034	377.1012
6-Aldehydo-7-methoxy-isoophiopogonone B	354.11034	377.1011			
3',5',β-Trihydroxy-3,4,4',α-tetramethoxychalcone	376.11582	415.0775			
			3-Hydroxy-5,7,8,3',4'-pentamethoxy flavone	388.11582	427.0771
			Gardenin E (flavone)	390.09508	413.0835
Sophoranodichromane D	408.19367	447.1587	Sophoranodichromane B (flavonoid)	408.19367	447.1586
			Liquiritin (flavanone glycoside)	418.12638	441.1155
			Mangiferin (glucosylxanthone)	422.08491	423.0907
			Ononin (4'-methoxyisoflavone)	430.12638	469.0902
Apigenin-7-O-galactopyranoside	432.10565	455.0945	Apigenin-7-O-galactopyranoside (flavone)	432.10565	455.0946
5,7,4′-Trihydroxy-8-C-β-D-flavanone glucoside	434.1213	435.1265			
			7-O-β-D-Glucopyrano-syl-kaempferol	448.10056	449.1059
Kaempferol-3-O-β-D-glucopyranoside	448.10056	471.0889	Kaempferol-3-O-β-D-glucopyranoside	448.10056	471.0895

			(2S)-5,7-Dihydroxy-6-methoxy-flavanone-7-O-β-D glucopyranoside	448.13695	487.1021
Homoplantaginin	462.11621	485.1044	Homoplantaginin (flavonoid)	462.11621	485.1051
			9,10-Dimethoxy-pterocarpan-3-O-β-D-glucoside (isoflavonoid)	462.1526	463.1577
Homoeriodictyol-7-O-β-D-glucopyranoside (flavonone)	464.13186	465.1369			
			Quercimeritrin (flavonol glucoside)	464.09548	487.0834
Pelargonidin 3-glucoside	468.0794	469.0867			
Hibiscetin-3-O-glucoside (red pigment)	496.08531	497.0947			
Peonidin 3-glucoside (red & purple)	498.09289	499.0981			
			Mirificin (daidzein 8-C-(6-apiofuranosylglucoside), is an isoflavone)	548.15299	587.1136
Isocryptomerin (biflavonoid)	552.10565	575.093			
			Apiin (diglycoside of the flavone apigenin)	564.14791	565.1528
Isoschaftoside (C-glycosyl compound that is apigenin)	564.14791	565.1529			
			5′-Methoxy-bilobetin (flavonoid)	582.11621	605.106
Neomangiferin (C-glucosyl xanthone)	584.13773	585.1429			
			Apigenin-7-O-α-L-rhamnose(1→4)-6''-O-acetyl-β-D-glucoside	620.17412	621.1792
Isorhamnetin-3-O-neohesperidoside	624.16903	663.1298	Isorhamnetin-3-O-neohesperidoside	624.16903	663.1309
Complanatuside (Rhamnocitrin is a monomethoxyflavone)	624.16903	663.1298			
			Delphinidin-3,5-diglucoside	625.14047	648.1206
Kaempferide-3-O-α-L-(4-O-acetyl)-rhamnosyl-7-O-α-L-rhamnoside	634.18977	657.1817			
			Malvidin-3-O-(6-O- acetyl-β-D-glucopyranoside)-5-O-β-D-glucopyranoside	696.19016	735.1492
			Vaccarin (Isovitexin 4'-O-glucoside 2''-O-arabinoside)	726.20073	727.2053
**Phenolics & their derivatives**			**Phenolics & their derivatives**		
Bergenin (trihydroxybenzoic acid)	328.07943	351.0677	Bergenin	328.07943	351.0682
			Meliadanoside B (phenylpropanetriol glycoside)	328.11582	351.1041

			Pseudolaroside B (3-methoxy-benzoic acid 4-O-b- D-allopyranoside)	330.09508	353.0839
			Citrusin C (phenolic glycoside)	342.16785	365.1577
4-Hydroxy ginkgolic acid (phenolic)	362.24571	401.2084	4-Hydroxy ginkgolic acid	362.24571	401.21
			3-O-trans-Feruloylquinic acid	368.11073	391.098
			Amurenamide A (phenolic)	385.13728	408.1259
			Polydatin (phenolic)	390.13147	429.0931
Sibirioside A	472.15808	495.1471	Sibirioside A (phenylpropanoid glycoside)	472.15808	495.1469
Blestritin B	486.20424	525.1667			
			2,4,6-Trihydroxyacetophenone-2,4-di-O-β-D-glucopyranoside	492.14791	515.137
			Indigoticoside A (phenolic glycoside)	522.21011	545.1994
Picroside III (Phenolic glycoside)	538.16864	561.1566	Picroside III	538.16864	577.134
Lithospermic acid (phenolic)	538.11113	539.119			
Monomethyl lithospermate	552.12678	591.0915			
			Erigoster A (phenolic)	558.13734	559.1431
			2,3,5,4'-Tetrahydroxystilbene-2-O-(6''-O-α-D-glucopyranosyl)-β-D-glucopyranoside (phenolic)	568.17921	569.1872
			Isocrenatoside (phenolic glycoside)	622.18977	661.1511
			Agrimol E (poly phenol)	626.23633	627.2406
			Lithospermic acid B	718.15338	719.1605
			ViscumneosideV(phenolic glycoside)	728.21638	729.2257
Parishin B (phenolic glucosides consisting of one molecule of citric acid)	728.21638	729.224			
**Tannins**			**Tannins**		
3,4-Dihydroxyphenothyl-3-O-β-D-glucopyranoside	288.08452	289.0909	3,4-Dihydroxyphenothyl-3-O-β-D-glucopyranoside	288.08452	289.091
Ellagic acid	302.00627	303.0127	Ellagic acid	302.00627	303.0126
			1-Galloyl-glucose	332.07435	355.0627
2,3-(S)-Hexahydroxydiphenoyl-D-glucose	482.06966	483.0759	2,3-(S)-Hexahydroxydiphenoyl-D-glucose (tannin)	482.06966	505.0594
			3,6-Di-O-Galloyl-β-D-glucose	484.08531	507.075
Bistortaside (tannin)	512.11661	513.1222			

Corilagin_1 (ellagitannin)	634.08061	657.0703	Corilagin_1	634.08061	657.0701
			Castalagin	934.07123	935.0812
**Lignins**			**Lignins**		
			Lignan	464.16825	465.1747
			Silymonin (flavanolignan)	466.12638	467.1359
			Dehydrosilybin (flavonolignan)	480.10565	481.1146
Silybin	482.1213	483.13	Silybin (flavonolignan)	482.1213	483.13
			Isosilybin	496.13695	497.1463
			(+)-1-Hydroxypinoresinol 4',4''-di-O-β-D-glucopyranoside(Pinoresinol diglucoside, tetrahydrofuran lignan)	698.2422	737.2069

Fresh berry vinegar (negative ion)			Dried berry vinegar (negative ion)		
**Compounds**	**Neutral mass (Da)**	**Observed m/z**	**Compounds**	**Neutral mass (Da)**	**Observed m/z**
**Organic acid and its derivatives**			**Organic acid and its derivatives**		
			Phenylacetic acid	136.05243	135.0445
			E-p-coumatic acid	164.04734	163.0396
Shikimic acid	174.05282	173.0452	Shikimic acid	174.05282	173.045
Citric acid	192.027	191.0192	Citric acid	192.027	191.0192
Quinic acid	192.06339	191.0555	Quinic acid	192.06339	191.0555
1,5-Dimethyl citrate	220.0583	219.0506	1,5-dimethyl citrate	220.0583	219.0503
			3-O-trans-coumaroylquinic acid	338.10017	337.0929
Chlorogenic acid	354.09508	353.0874	Chlorogenic acid	354.09508	353.0875
3-O-trans-Feruloylquinic acid	368.11073	367.1051			
Methyl 3-O-feruloylquinate	382.12638	381.1189	Methyl 3-O-feruloylquinate	382.12638	381.119
Propyl chlorogenate	396.14203	395.1343	Propyl chlorogenate	396.14203	395.1345
**Flavonoids & its derivatives**			**Flavonoids & its derivatives**		
Cyanidin	322.02442	321.0135	Cyanidin	322.02442	321.0135
			3,5,6-Trihydroxy-4',7-dimethoxyflavone	330.07395	329.0673
Eupatin (3,3',5-trihydroxy-4',6,7-trimethoxyflavone)	360.08452	359.079			

			Quercetin-3-sulphate	381.99947	380.991
Mangiferin	422.08491	421.0759			
Kaempferol-3-O-β-D-glucopyranoside	448.10056	447.093	Kaempferol-3-O-β-D-glucopyranoside	448.10056	447.0933
5,7,2′-Trihydroxy-flavanone-4′-O-β-D-glucoside	450.11621	449.1087	5,7,2′-Trihydroxy-flavanone-4′-O-β-D-glucoside	450.11621	449.1088
6-Hydroxykaempferol-3-O-glucoside	464.09548	463.0878	6-Hydroxykaempferol-3-O-glucoside	464.09548	463.0879
Quercimeritrin (Quercetin-7-O-β-D-glucopyranoside)	464.09548	463.088	Quercimeritrin	464.09548	463.0882
3-O-β-D-Galacopyanosyl quercetin	466.11113	465.1041	3-O-β-D-Galacopyanosyl quercetin	466.11113	465.1041
Pelargonidin 3-glucoside	468.08232	467.0719			
			Isosilybin (flavonolignan)	496.13695	495.1318
			Hibiscetin-3-O-glucoside	496.08531	495.0814
Peonidin 3-glucoside	498.09289	497.0894			
			Neocomplanoside (flavonoid)	504.12678	503.1214
Sotetsuflavone	552.10565	551.0994	Sotetsuflavone	552.10565	551.0969
Neomangiferin	584.13773	583.1284	Neomangiferin (xanthone)	584.13773	583.1289
Malvidin 3,5-diglucoside	654.1796	653.1799			
Delphin	662.12498	661.1236			
**Phenolics and its derivatives**			**Phenolics and its derivatives**		
Methyl-3-hydroxy-4-methoxybenzoate	182.05791	181.0501	Methyl-3-hydroxy-4-methoxybenzoate	182.05791	181.0501
3-Ethoxy-4,5-dihydroxybenzoic acid	198.05282	197.045	3-Ethoxy-4,5-dihydroxybenzoic acid	198.05282	197.045
Brevifolin	248.03209	247.0241	Brevifolin (acetophenone)	248.03209	247.0242
			Norbergenin (O-demethylated derivative of bergenin, trihydroxybenzoic acid)	314.06378	313.0563
Meliadanoside B	328.11582	327.1083	Meliadanoside B (phenylpropanetriol glycoside)	328.11582	327.108
Vanillic acid β-D-glucopyranosyl ester	330.09508	329.0873			
Polygoacetophenoside (acetophenone glucoside, a sweet pungent taste and odor resembling the odor of orange)	346.09	345.0829			
Cistanoside F (phenylethanoid glycoside)	488.15299	487.146	Cistanoside F	488.15299	487.1457
2,4,6-Trihydroxyacetophenone-2,4-di-O-β-D-glucopyranoside	492.14791	491.1408	2,4,6-Trihydroxyacetophenone-2,4-di-O-β-D-glucopyranoside	492.14791	491.1407
Curculigine B (chlorinated phenol glycoside)	500.08522	499.0797			
Indigoticoside A (phenolic glycoside)	522.21011	521.203	Indigoticoside A	522.21011	521.2031

Furosin (cyclic & phenolic, Maillard products)	650.07553	649.069	Furosin	650.07553	649.0689
			Shegansu A (phenylpropanoid ester of sucrose)	672.16903	671.1604
Suffruticosol A (poly phenolic)	680.20463	679.1965			
			Tubuloside C (phenolic glycoside)	954.3005	953.2885
**Tannins**			**Tannins**		
Gallic acid	170.02152	169.0136	Gallic acid	170.02152	169.0136
Ellagic acid	302.00627	300.9984			
1-Galloyl-β-D-glucose	332.07435	331.0668	1-Galloyl-β-D-glucose	332.07435	331.0668
			2,6-Bis(4-hydroxyphenyl)-3′,5-dimethoxy-3-hydroxybibenzyl	470.20932	469.2035
2,3-(S)-Hexahydroxydiphenoyl-D-glucose	482.06966	481.0629	2,3-(S)-Hexahydroxydiphenoyl-D-glucose	482.06966	481.0624
3,6-Di-O-Galloyl-β-D-glucose	484.08531	483.078	3,6-Di-O-Galloyl-β-D-glucose	484.08531	483.078
			Nilocitin (2,3-digalloyl-D-glucopyranose)	484.08531	483.078
Corilagin_1 (ellagitannin)	634.08061	633.073	Corilagin_1	634.08061	633.0744
Pedunculagin	784.07592	783.0697	Pedunculagin (ellagitannin)	784.07592	783.0713
Castalagin	934.07123	933.0667	Castalagin	934.07123	933.0652
